# Supplementary material for: Mapping the evolution of fertility support policies in China: A content and instrumental analysis
Source: PLoS One. 2025 Oct 9;20(10):e0332137. doi: 10.1371/journal.pone.0332137 (PMC12510515; doi:10.1371/journal.pone.0332137)
Supplement: S1 Appendix — (ZIP) [file pone.0332137.s001.zip › S1 Appendix. 226 original policy documents/203-第十三届全国人民代表大会第一次会议关于2017年中央和地方预算执行情况与2018年中央和地方预算的决议(FBM-CLI-1-311594).doc]

第十三届全国人民代表大会第一次会议关于2017年中央和地方预算执行情况与2018年中央和地方预算的决议

**发布部门：** [全国人民代表大会](https://www.pkulaw.com/law/chl?Aggs.IssueDepartment=101)

**发布日期：**2018.03.20

**实施日期：**2018.03.20

**时效性：** [现行有效](https://www.pkulaw.com/law/chl?Aggs.TimelinessDic=01)

**效力级别：** [工作文件](https://www.pkulaw.com/law/chl?Aggs.EffectivenessDic=XA0105)

**法规类别：** [人大议事](https://www.pkulaw.com/law/chl?Aggs.Category=00106) [预算管理](https://www.pkulaw.com/law/chl?Aggs.Category=03126) [营商环境优化](https://dobusiness.pkulaw.com/)

第十三届全国人民代表大会第一次会议关于2017年中央和地方预算执行情况与2018年中央和地方预算的决议
（2018年3月20日第十三届全国人民代表大会第一次会议通过）

　　第十三届全国人民代表大会第一次会议审查了国务院提出的《关于2017年中央和地方预算执行情况与2018年中央和地方预算草案的报告》及2018年中央和地方预算草案，同意全国人民代表大会财政经济委员会的审查结果报告。会议决定，批准《关于2017年中央和地方预算执行情况与2018年中央和地方预算草案的报告》，批准2018年中央预算。

关于2017年中央和地方预算执行情况与2018年中央和地方预算草案的报告
--2018年3月5日在第十三届全国人民代表大会第一次会议上
财政部

各位代表：
　　受国务院委托，现将2017年中央和地方预算执行情况与2018年中央和地方预算草案提请十三届全国人大一次会议审议，并请全国政协各位委员提出意见。
　　**一、**2017年中央和地方预算执行情况
　　2017年，在以习近平同志为核心的党中央坚强领导下，各地区各部门牢固树立政治意识、大局意识、核心意识、看齐意识，以习近平新时代中国特色社会主义思想为指导，全面深入贯彻党的十九大精神，坚持稳中求进工作总基调，坚持新发展理念，坚持以提高发展质量和效益为中心，以推进供给侧结构性改革为主线，严格执行十二届全国人大五次会议审查批准的预算，认真落实全国人大财政经济委员会的审查意见，统筹推进稳增长、促改革、调结构、惠民生、防风险各项工作，经济运行稳中向好态势持续发展，改革开放全面深化，经济结构继续优化，质量效益明显提升，风险管控稳步推进，市场信心显著增强，社会大局保持稳定。中央和地方预算执行情况较好。
　　（一）2017年一般公共预算收支情况。
　　1.全国一般公共预算。
　　全国一般公共预算收入172566.57亿元，为预算的102.3%，比2016年同口径（下同）增长7.4%。加上使用结转结余及调入资金10138.85亿元（包括中央和地方财政从预算稳定调节基金、政府性基金预算、国有资本经营预算调入资金，以及地方财政使用结转结余资金），收入总量为182705.42亿元。全国一般公共预算支出203330.03亿元，完成预算的104.3%，增长7.7%。加上补充中央预算稳定调节基金3175.39亿元，支出总量为206505.42亿元。收支总量相抵，赤字23800亿元，与预算持平。
　　2017年全国一般公共预算收入扭转了2012年以来增速放缓态势。一些收入项目超出预算较多，其中，国内增值税超出2677.56亿元，企业所得税超出1875.61亿元，进口货物增值税、消费税和关税超出3086.25亿元。主要是供给侧结构性改革深入推进，经济发展的质量和效益提升，税源相应增加；工业生产者价格指数回升超出预期，直接带动以现价计算的相关税收快速增长；国内外需求回暖，进口商品量价齐增，进口税收增加较多。
　　2.中央一般公共预算。
　　中央一般公共预算收入81119.03亿元，为预算的103.2%，增长7.1%。加上从中央预算稳定调节基金调入1350亿元，从中央政府性基金预算、中央国有资本经营预算调入283.35亿元，收入总量为82752.38亿元。中央一般公共预算支出95076.99亿元（包括本级支出、对地方税收返还和转移支付），完成预算的99.3%，增长5.4%。加上补充中央预算稳定调节基金3175.39亿元，支出总量为98252.38亿元。收支总量相抵，中央财政赤字15500亿元，与预算持平。
　　主要收入项目执行情况。国内增值税28165.97亿元，为预算的104.9%。国内消费税10225.09亿元，为预算的99.6%。进口货物增值税、消费税15968.56亿元，为预算的120.8%。关税2997.69亿元，为预算的112.7%。企业所得税20422.62亿元，为预算的104.4%。个人所得税7180.71亿元，为预算的111.2%。出口货物退增值税、消费税13870.37亿元，为预算的106.7%。非税收入5424.43亿元，为预算的66.2%。
　　中央本级支出29858.89亿元，完成预算的100.9%，增长7.5%。主要支出项目执行情况是：一般公共服务支出1271.46亿元，完成预算的100.9%。外交支出519.67亿元，完成预算的95.2%。国防支出10226.35亿元，完成预算的100%。公共安全支出1848.94亿元，完成预算的100.6%。教育支出1548.39亿元，完成预算的101.9%。科学技术支出2826.96亿元，完成预算的99.5%。粮油物资储备支出1597.48亿元，完成预算的108.2%。债务付息支出3779.43亿元，完成预算的100.8%。
　　中央对地方税收返还和转移支付65218.1亿元，完成预算的99.3%，增长4.5%。其中，税收返还8163.59亿元，完成预算的89.3%，主要是根据全面推开营改增试点后有关税收返还政策据实安排；一般性转移支付35167.9亿元，完成预算的100.4%；专项转移支付21886.61亿元，完成预算的101.9%。一般性转移支付占转移支付总额的比重提高至61.6%，其中，老少边穷地区转移支付增长19.7%，县级基本财力保障机制奖补资金增长9.5%。
　　2017年中央一般公共预算收入超收2507.38亿元、支出结余668.01亿元，全部转入中央预算稳定调节基金。中央预备费预算500亿元，实际支出60.7亿元，用于大气污染成因与治理攻关等方面，剩余439.3亿元（已包含在上述支出结余668.01亿元中）全部转入中央预算稳定调节基金。2017年末，中央预算稳定调节基金余额4666.05亿元。
　　3.地方一般公共预算。
　　地方一般公共预算收入156665.64亿元，其中，地方一般公共预算本级收入91447.54亿元，增长7.7%；中央对地方税收返还和转移支付收入65218.1亿元。加上地方财政使用结转结余及从地方预算稳定调节基金、政府性基金预算、国有资本经营预算调入资金8505.5亿元，收入总量为165171.14亿元。地方一般公共预算支出173471.14亿元，增长7.7%。收支总量相抵，地方财政赤字8300亿元，与预算持平。
　　（二）2017年政府性基金预算收支情况。
　　全国政府性基金收入61462.49亿元，增长34.8%。加上2016年结转收入298.5亿元和地方政府发行专项债券筹集收入8000亿元，全国政府性基金相关收入总量为69760.99亿元。全国政府性基金相关支出60700.22亿元，增长32.7%。
　　中央政府性基金收入3824.77亿元，为预算的103.2%，增长6.4%。加上2016年结转收入298.5亿元，中央政府性基金收入总量为4123.27亿元。中央政府性基金支出3669.19亿元，完成预算的91.6%，增长9.2%。其中，中央本级支出2683.6亿元，对地方转移支付985.59亿元。中央政府性基金收大于支454.08亿元。其中，结转下年继续使用385.59亿元；单项政府性基金结转超过当年收入30%的部分合计68.49亿元，按规定补充中央预算稳定调节基金。
　　地方政府性基金本级收入57637.72亿元，增长37.3%，其中国有土地使用权出让收入52059.01亿元，增长40.7%。加上中央政府性基金对地方转移支付收入985.59亿元和地方政府发行专项债券筹集收入8000亿元，地方政府性基金相关收入为66623.31亿元。地方政府性基金相关支出58016.62亿元，增长34.2%，其中国有土地使用权出让收入相关支出51779.63亿元，增长37.1%。
　　（三）2017年国有资本经营预算收支情况。
　　国有资本经营预算收入主要根据国有企业上年实现净利润一定比例收取，同时按照收支平衡原则安排相关支出。
　　全国国有资本经营预算收入2578.69亿元，下降1.2%。全国国有资本经营预算支出2010.93亿元，下降6.7%。
　　中央国有资本经营预算收入1244.27亿元，为预算的96.5%，下降13%，主要是石油、电力等行业企业2016年经济效益下滑。加上2016年结转收入128.03亿元，收入总量为1372.3亿元。中央国有资本经营预算支出1001.71亿元，完成预算的86.3%，下降30.9%，主要是2017年国有资本经营预算收入规模下降，同时调入一般公共预算比例提高，支出规模相应减少。其中，中央本级支出766.34亿元，对地方转移支付235.37亿元。向一般公共预算调出257亿元。结转下年支出113.59亿元。
　　地方国有资本经营预算本级收入1334.42亿元，增长13.2%。加上中央国有资本经营预算对地方转移支付收入235.37亿元，收入总量为1569.79亿元。地方国有资本经营预算支出1244.59亿元，增长2.1%。向一般公共预算调出310.61亿元。
　　（四）2017年社会保险基金预算收支情况。
　　全国社会保险基金收入55380.16亿元，为预算的106.9%，增长10.5%。其中，保险费收入39563.61亿元，财政补贴收入12264.49亿元。全国社会保险基金支出48951.67亿元，完成预算的101%，增长12.3%。当年收支结余6428.49亿元，年末滚存结余72037.47亿元。目前全国社会保险基金整体上还有较大结余，但受人口老龄化等因素影响，部分省份企业职工基本养老保险抚养比偏低，基金收支平衡压力较大。
　　2017年末，中央财政国债余额134770.16亿元，控制在全国人大批准的债务余额限额141408.35亿元以内；地方政府债务余额164706.59亿元，包括一般债务余额103322.35亿元、专项债务余额61384.24亿元，控制在全国人大批准的债务余额限额188174.3亿元以内。
　　以上预算执行的具体情况详见《中华人民共和国2017年全国预算执行情况2018年全国预算（草案）》。
　　（五）2017年主要财税政策落实和重点财政工作情况。
　　2017年，财政部门认真落实十二届全国人大五次会议预算决议要求，全面贯彻实施[预算法](https://www.pkulaw.com/chl/0b971dd30ea57e0ebdfb.html)，有效实施积极的财政政策，持续深化财税体制改革，加大重点领域保障力度，支持推进供给侧结构性改革，有力促进了经济社会持续健康发展。
　　着力实施减税降费政策。密切跟踪营改增试点实施情况，落实并不断完善试点政策，简化增值税税率结构。享受减半征收企业所得税优惠的小型微利企业年应纳税所得额上限由30万元提高到50万元。科技型中小企业研发费用加计扣除比例由50%提高到75%。启动创业投资和天使投资有关税收政策试点。将商业健康保险个人所得税税前扣除试点政策推至全国。继续实施2016年底到期的部分税收优惠政策等。实行上述措施，全年新增减税超过3800亿元。取消、停征或免征房屋转让手续费等43项中央行政事业性收费，降低7项收费标准；取消、停征或减免城市公用事业附加等5项政府性基金，降低2项征收标准；取消工业企业结构调整专项资金。加上地方自主清理的政府性收费，全年涉及减收超过1900亿元。此外，相关部门还出台了清理规范经营服务性收费、推进网络提速降费、降低企业用能成本、推进物流降本增效等措施，全年减轻社会负担超过4400亿元。进一步规范涉企收费行为，创建实施政府性基金和行政事业性收费、[政府定价的经营服务性收费目录清单](https://www.pkulaw.com/chl/5a4c445dc7c812b8bdfb.html)全国“一张网”制度，清单之外一律不得收费，并建立了乱收费举报投诉查处机制。
　　扎实推进供给侧结构性改革。落实“三去一降一补”重点任务。拨付专项奖补资金222亿元，支持钢铁、煤炭行业超额完成2017年化解过剩产能任务，妥善安置分流职工。淘汰停建缓建煤电产能6500万千瓦。拨付资金566.7亿元，支持中央企业处置“僵尸企业”以及解决国有企业“三供一业”等历史遗留问题。完善棚改安置方式，在商品房库存量大的地方积极推行货币化安置。落实和完善企业兼并重组、债权转让核销等相关政策，市场化法治化债转股工作有序推进。进一步优化中央基建投资支出结构，集中用于重大水利工程、新一轮农网改造升级等。规范推行政府和社会资本合作（PPP）模式，民营企业参与率稳步提高。推进农业供给侧结构性改革。启动41个国家现代农业产业园创建。实施农业补贴制度改革，突出绿色生态导向。支持完成粮改饲面积超过1300万亩，耕地轮作休耕制度试点1200万亩，重金属污染耕地修复治理242万亩，引导种植结构调整，促进耕地地力保护。完善粮食等重要农产品价格形成机制和收储制度，消化政策性粮棉油库存8501万吨。国家农业信贷担保联盟有限公司和33家省级农业信贷担保公司完成组建，累计为超过4.9万个农业项目提供贷款担保，担保总额442.5亿元，缓解了农业经营主体融资难、融资贵、融资慢问题。在13个粮食主产省选择200个产粮大县，面向适度规模经营农户开展农业大灾保险试点，保障水平提高约90%。推进土地整治，农业综合开发建设高标准农田2500万亩，高效节水灌溉面积新增2165万亩，灾后水利薄弱环节和重大水利基础设施建设有序推进。支持18个省份开展田园综合体建设试点示范。
　　培育壮大经济发展新动能。加强对公共科技活动的支持，全面落实中央财政科技计划和科研项目资金管理改革举措，运用市场化方式促进科技成果转移转化。深入实施“中国制造2025”，支持智能制造、工业强基、绿色制造工程建设。落实和完善首台（套）重大技术装备保险补偿政策，将新材料纳入首批次应用保险保费补偿范围。发挥国家集成电路产业投资基金等引导作用，促进制造业重点领域加快发展。实施调整后的财政补贴政策，全年推广新能源汽车约80万辆。支持30个城市开展小微企业创业创新基地城市示范，加快国家中小企业发展基金运行，促进大众创业、万众创新蓬勃发展。
　　大力支持脱贫攻坚。中央财政补助地方专项扶贫资金861亿元，比2016年增长30.3%，农业、教育、医疗、交通等领域也加大了对贫困地区的投入力度。在全国832个国家扶贫开发工作重点县和连片特困地区县全面推开涉农资金整合试点，截至2017年底，实际整合财政涉农资金3286亿元。中央财政安排易地扶贫搬迁贷款贴息，支持地方完成340万建档立卡贫困人口易地扶贫搬迁建设任务。大力推进资产收益扶贫，带动贫困群众增收。脱贫攻坚继续保持良好态势，全年实现1289万农村贫困人口脱贫。
　　保障和改善民生。促进教育改革发展。统一城乡义务教育学生“两免一补”政策，全国约1.43亿义务教育学生获得免学杂费、免费教科书资助，1377万家庭经济困难寄宿生获得生活补助，1400万进城务工农民工随迁子女实现相关教育经费可携带。落实高等教育领域“放管服”改革要求，完善和加强高校经费使用管理，进一步扩大高校办学自主权。博士生国家助学金资助标准提高了3000元。深化医药卫生体制改革。全面推开公立医院综合改革，取消了实行60多年的药品加成政策。开展生育保险和职工基本医疗保险合并实施试点。城乡居民基本医疗保险财政补助标准由每人每年420元提高到450元。基本公共卫生服务项目年人均财政补助标准由45元提高到50元。整合公共卫生服务项目，改革资金分配方式。推进基层卫生综合改革。支持做好就业和社会保障工作。完善就业创业政策体系，扩大求职创业补贴补助范围，鼓励地方对符合条件的高校毕业生、就业困难人员创业给予一次性补贴。出台划转部分国有资本充实社保基金实施方案。机关事业单位和企业退休人员基本养老金标准提高了约5.5%。整合设立困难群众救助补助资金，由各地统筹用于低保、特困人员救助供养、临时救助、流浪乞讨人员救助、孤儿基本生活保障支出，惠及全国7797万困难群众。提高优抚对象等人员抚恤和生活补助标准，惠及全国860万优抚对象。完善救灾补助政策，大幅提高灾害应急救助等4项中央财政补助标准。加强基本住房保障。支持棚户区改造开工609万套，推进公租房配套基础设施建设。中央财政农村危房改造补助资金集中用于低保户、农村分散供养特困人员、贫困残疾人家庭和建档立卡贫困户等4类重点对象，户均补助标准由2016年7500元（贫困地区为8500元）大幅提高至2017年约1.4万元，支持改造农村危房190.6万户。推动文化体育事业发展。加快构建现代公共文化服务体系，49863家公益性文化设施向社会免费开放，1257个体育馆向社会免费或低收费开放，城乡公共文化体育基础条件进一步改善。
　　支持生态环保建设。落实京津冀及周边、长三角、珠三角地区大气污染防治重点任务。启动中央财政支持北方地区冬季清洁取暖试点。支持地方落实水污染、土壤污染防治行动计划。增加国家重点生态功能区转移支付规模，全国享受重点补助的县域达819个。加大祁连山生态环境保护支持力度，推动妥善解决历史遗留问题。开展第二批山水林田湖草生态保护和修复工程试点。推进湿地保护与恢复，落实新一轮退耕还林还草、新一轮草原生态保护补助奖励和天然林保护全覆盖政策，支持全面停止天然林商业性采伐。支持循环经济发展和清洁生产，启动畜禽粪污资源化利用试点。深入开展排污权有偿使用和交易试点。完善流域上下游横向生态保护补偿机制。支持建立国家公园体制试点。
　　深化财税体制改革。改进预算管理制度。清理规范重点支出同财政收支增幅或生产总值挂钩事项，完善重点支出保障机制。财政转移支付结构进一步优化，专项转移支付项目数量减少18项、减至76项。首次在中国政府网、财政部门户网站集中公开中央政府预算、中央部门预算和中央对地方转移支付，公开部门预算的中央部门增加到105个。完善政府预算体系，中央国有资本经营预算调入一般公共预算的比例提高到22%，将新增建设用地土地有偿使用费等3项政府性基金项目转列一般公共预算，取消排污费等以收定支、专款专用的规定。强化预算绩效管理，绩效目标管理范围覆盖中央部门所有一级和二级项目、全部中央对地方专项转移支付，绩效目标随同预算批复和下达，全面开展项目绩效目标执行监控和绩效自评，会同第三方机构开展重点绩效评价，部分绩效自评结果和重点绩效评价结果向全国人大报告并向社会公开。健全政府会计准则体系，开展政府财务报告编制试点。推进税制改革和税收立法。完善增值税制度，将增值税税率由四档减至17%、11%和6%三档，取消13%的税率，将农产品等税率从13%降至11%。水资源税改革试点扩大到10个省份。出台个人收入和财产信息系统建设总体方案。配合加快税收立法工作，[烟叶税法](https://www.pkulaw.com/chl/41b156c3ff1e780cbdfb.html)、[船舶吨税法](https://www.pkulaw.com/chl/8225cc2614100d90bdfb.html)以及[环境保护税法](https://www.pkulaw.com/chl/78d5d4163c8fba53bdfb.html)实施条例顺利出台，[增值税暂行条例](https://www.pkulaw.com/chl/ea435225c018eaaebdfb.html)修订公布，耕地占用税法、车辆购置税法、资源税法、关税法等立法工作深入开展。完善财政体制。分领域推进中央与地方财政事权和支出责任划分改革，出台外交领域改革方案，起草教育、医疗卫生领域改革方案。研究进一步理顺中央与地方收入划分改革总体方案，起草健全地方税体系改革方案。
　　加强地方政府债务管理。依法规范地方政府举债融资，强化地方政府债务限额管理，全年发行地方政府新增债券1.59万亿元，控制在全国人大批准的2017年新增地方政府债务限额1.63万亿元以内。着力发展项目收益与融资自求平衡的地方政府专项债券品种，启动发行土地储备、政府收费公路专项债券。印发进一步规范地方政府举债融资行为、坚决制止地方以政府购买服务名义违法违规融资等文件，设定“正面清单”和“负面清单”，严禁各种形式的违法违规或变相举债，初步实现对当前地方政府主要违法违规融资行为的监管政策全覆盖。组织核查部分市县和金融机构违法违规融资担保行为，严格依法依规问责处理，相关责任人被给予撤职、行政降级、罚款等处分。公开曝光处理结果，发挥警示教育作用。同时，继续开展地方政府债务风险评估和预警，督促指导地方有效防范政府债务风险。
　　强化财政监督。组织开展地方预决算公开、扶贫资金使用管理、中央部门财经纪律执行情况、工业企业结构调整专项奖补资金等监督检查，查处违规使用财政资金490多亿元，对相关责任人严肃追责，推动完善制度、规范管理，促进重大财税政策和制度落实。
　　2017年的财政预算工作，是过去五年财政改革发展实践的重要组成部分。五年来，国家财政实力不断迈上新台阶，财政政策的精准性有效性不断实现新提升，现代财政治理体系和能力建设不断取得新突破，财政在党和国家事业取得历史性成就、发生历史性变革的伟大进程中发挥了积极作用。一是坚持实施积极的财政政策，促进经济运行保持在合理区间。全国一般公共预算支出由2012年的12.6万亿元增加到2017年的20.3万亿元，赤字率一直控制在3%以内。全面推开营改增试点，实现增值税对货物和服务全覆盖，征收66年的营业税退出历史舞台，营改增累计减税超过2万亿元。取消、免征、停征或减征1368项政府性基金和行政事业性收费，涉及减收金额3845亿元。积极调整财政支出结构、盘活沉淀资金，保障基本民生和重点项目。二是坚持推进供给侧结构性改革，推动实现供求关系新的动态均衡。扎实推进“三去一降一补”，出台实施一批有利于实体经济健康发展的财税政策，促进经济结构优化和新动能加快成长。着眼于提高农业供给质量，进一步完善财政支农政策，支持构建现代农业产业体系、生产体系、经营体系，推动农业农村发展向绿色生态可持续、更加注重满足质的需求转变。深入实施创新驱动发展战略，持续加大财政科技投入力度、优化投入结构、完善支持方式，创新对发展的支撑作用不断增强。三是坚持深化财税体制改革，现代财政制度建设取得重要阶段性成果。在推进预算公开、实行中期财政规划管理、完善转移支付制度、加强预算绩效管理等方面，出台实施一系列重大改革举措，现代预算管理制度主体框架基本建立。完善消费税制度，调整征收范围、优化税率结构和改进征税环节。全面推进资源税从价计征改革。加快推进财政事权和支出责任划分改革。合理确定全面推开营改增试点后增值税收入分享比例，中央和地方财力格局总体稳定。四是坚持保障和改善民生，人民群众获得感不断增强。深入实施精准扶贫、精准脱贫，中央财政补助地方专项扶贫资金年均增长22.7%。建立城乡统一、重在农村的义务教育经费保障机制，健全家庭经济困难学生资助政策体系。调整完善就业创业政策措施，重点群体和总体就业形势保持稳定。基本医保制度实现全覆盖，大病保险制度基本建立，城乡居民基本养老保险制度实现统一。构建起以低保、特困人员救助供养、临时救助等为主体的社会救助制度体系。连续提高退休人员基本养老金、城乡低保、优抚等标准。全国城镇保障性安居工程开工建设3400多万套，完成农村危房改造1700多万户。五是坚持推进依法理财，提升财政管理水平。修改后的[预算法](https://www.pkulaw.com/chl/0b971dd30ea57e0ebdfb.html)颁布施行，出台[环境保护税法](https://www.pkulaw.com/chl/78d5d4163c8fba53bdfb.html)、[资产评估法](https://www.pkulaw.com/chl/948190a243a1996bbdfb.html)、[政府采购法实施条例](https://www.pkulaw.com/chl/22116378f068aa3dbdfb.html)等，财税法治建设不断取得新进展。全面推行“双随机、一公开”监管，加大财政监督检查力度。建立规范的地方政府举债融资机制，强化地方政府债务管理，初步形成地方政府债务监督管理制度框架和风险防控体系。现代国库管理体系建设向纵深推进，国债市场化改革、收益率曲线建设取得重要成果，政府采购运行机制不断优化。建立健全财政部和财政系统内控机制，财政核心业务及权力运行的风险管控与监督制衡持续加强。配合推进预算联网监督，中央预算联网监督系统建成并上线运行，省级财政部门全面实现与同级人大联网，主动接受人大监督。
　　五年来财政改革发展工作取得的成绩，是以习近平同志为核心的党中央坚强领导的结果，是习近平新时代中国特色社会主义思想科学指引的结果，是全国人大、全国政协及代表委员们监督指导的结果，是各地区、各部门以及全国各族人民共同努力的结果。同时，我们也清醒地看到，预算执行和财政工作中还面临一些困难和挑战。主要是：财政收支紧平衡特征明显，部分基层财政运行困难，保工资、保运转、保基本民生面临较大压力；财政支出结构还需进一步优化，支出项目只增不减的固化格局没有根本改变，资金使用碎片化问题亟待破解；预算执行不够均衡，年末结转资金规模较大，部分专项转移支付项目预算执行率偏低；加强财政绩效管理十分紧迫，一些地方、部门和预算单位重分配轻管理，花钱不问效，资金闲置浪费问题严重；财税改革还有不少硬骨头，有的改革需要进一步加快，有的改革举措落地见效需要下更大功夫；一些地方违法违规或变相举债，风险隐患不容忽视。我们高度重视这些问题，将采取有力措施加以解决。

　　**二、**2018年中央和地方预算草案
　　2018年是贯彻党的十九大精神的开局之年，是改革开放40周年，是决胜全面建成小康社会、实施“十三五”规划承上启下的关键一年，做好预算编制和财政工作意义重大。要适应新时代、聚焦新目标、落实新部署，更好发挥财政在国家治理中的基础和重要支柱作用，推动我国经济在实现高质量发展上不断取得新进展。
　　（一）2018年财政收支形势分析。
　　财政收入方面。2018年，全面贯彻落实党的十九大精神将对深化改革、促进发展形成新的强大动力，我国经济持续健康发展具有许多有利条件。同时，国际环境和经济发展的不稳定性不确定性因素增多，财政增收也存在一些压力和挑战，主要是价格因素增收作用可能减弱，新老减税降费措施叠加将产生较大减收。财政支出方面。各级财政必保支出较多，新增支出需求很大。打好防范化解重大风险、精准脱贫、污染防治的攻坚战，需要优先保障。深化供给侧结构性改革、加快建设创新型国家、实施乡村振兴战略、加强国防和军队现代化建设、推进中国特色大国外交等，都需要重点支持。就业、教育、医疗、居住、养老等民生领域还有不少短板，需要加大投入。综合判断，2018年财政收入继续保持向好态势与财政支出快速增长并存，预算收支安排依然是紧平衡。各级财政要坚持过紧日子，勤俭节约、艰苦奋斗、精打细算，切实把财政资金用在增强发展后劲、让人民群众过好日子上。
　　（二）2018年预算编制和财政工作的指导思想和原则。
　　2018年预算编制和财政工作的指导思想是：在以习近平同志为核心的党中央坚强领导下，以马克思列宁主义、毛泽东思想、邓小平理论、“三个代表”重要思想、科学发展观、习近平新时代中国特色社会主义思想为指导，全面深入贯彻党的十九大和十九届二中、三中全会精神，贯彻党的基本理论、基本路线、基本方略，坚持和加强党的全面领导，坚持稳中求进工作总基调，坚持新发展理念，紧扣我国社会主要矛盾变化，按照高质量发展的要求，统筹推进“五位一体”总体布局和协调推进“四个全面”战略布局，坚持以供给侧结构性改革为主线，继续实施积极的财政政策，增强财政可持续性。继续实施减税降费政策，进一步减轻企业负担；调整优化支出结构，确保对重点领域和项目的支持力度，严控一般性支出，提高财政资金使用效率，着力支持在打好防范化解重大风险、精准脱贫、污染防治的攻坚战方面取得扎实进展，推动解决发展不平衡不充分问题；严格贯彻[预算法](https://www.pkulaw.com/chl/0b971dd30ea57e0ebdfb.html)，牢固树立过紧日子的思想，完善预算管理制度，全面实施绩效管理；建立权责清晰、财力协调、区域均衡的中央和地方财政关系，加快推进基本公共服务均等化，促进经济社会持续健康发展。
　　贯彻上述指导思想，要立足于我国经济已转向高质量发展阶段这一基本特征，着重把握好以下原则：一是继续实施减税降费。坚持把发展经济的着力点放在实体经济上，结合完善税制，适时出台新的减税降费措施，大力降低实体经济成本。二是调整优化支出结构。提高财政支出的公共性和普惠性，严控一般性支出，确保对供给侧结构性改革、脱贫攻坚、生态环保、教育、医疗卫生、国防等领域和重点项目的支持力度。三是深化财税体制改革。按照加快建立现代财政制度的要求，推进中央与地方财政事权和支出责任划分改革，提高预算约束力和透明度，完善税收制度，提升财政资源配置效率。四是促进区域协调发展。积极支持实施区域协调发展战略，发挥转移支付促进区域协调发展的作用，加快缩小地区间基本公共服务水平差距，增强困难地区和基层政府保工资、保运转、保基本民生的能力。五是全面实施绩效管理。预算安排与绩效管理相结合，将有限的财政资金用在刀刃上，以绩效为导向，严格支出管理，进一步提高资金使用效益。六是增强财政可持续性。合理安排收支预算和适当降低赤字率，为今后宏观调控拓展政策空间。坚决制止地方政府违法违规举债行为。做好民生工作，坚持尽力而为、量力而行。
　　（三）2018年财政政策。
　　2018年，积极的财政政策取向不变，要聚力增效。按照三档并两档的方向，调整增值税税率水平，重点降低制造业、交通运输等行业税率，优化纳税服务。实施个人所得税改革。统一小规模纳税人年销售额标准。再次扩大享受减半征收所得税优惠政策的小微企业范围。将创业投资和天使投资有关税收优惠政策试点范围扩大到全国。对企业新购入500万元以下的设备、器具当年一次性在税前扣除。实施企业境外所得综合抵免政策。扩大物流企业仓储用地税收优惠范围。继续实施部分2017年到期的税收优惠政策等。实行上述措施，预计全年再减税8000多亿元。加上继续阶段性降低企业“五险一金”缴费比例、进一步清理规范政府性基金和行政事业性收费等各种收费，将减轻税费负担1万亿元以上。同时，统筹收入、赤字、专项债务和调用预算稳定调节基金，适度扩大财政支出规模，为决胜全面建成小康社会、建设现代化经济体系、提高保障和改善民生水平等提供必要的财力保障。
　　2018年主要收支政策：
　　1.支持打好三大攻坚战。
　　支持打好防范化解重大风险攻坚战。坚持标本兼治、疏堵结合、循序渐进，积极防范化解地方政府债务风险。适度增加地方政府专项债券规模，优先支持地方在建项目平稳建设，2018年安排地方政府专项债券13500亿元，比2017年增加5500亿元。坚决遏制隐性债务增量，积极稳妥处置隐性债务存量，坚持谁举债、谁负责，严格落实地方政府属地管理责任，债务人、债权人依法合理分担风险。
　　支持打好精准脱贫攻坚战。在现行标准下，按照精准扶贫、精准脱贫基本方略，多渠道筹措资金，瞄准特定贫困群众精准帮扶，进一步向深度贫困地区聚焦发力。中央财政补助地方专项扶贫资金安排1060.95亿元，比2017年增加200亿元，增长23.2%，增量重点用于“三区三州”等深度贫困地区。加大教育转移支付、重点生态功能区转移支付、中央基建投资等资金渠道向深度贫困地区的倾斜力度。深入推进产业、教育、健康、生态和文化等扶贫，补齐基础设施和公共服务短板。严格按照规定的范围和用途，加快推进贫困县涉农资金实质性整合，全面实施扶贫资金绩效管理，建立完善常态化监管机制，切实管好用好扶贫资金。
　　支持打好污染防治攻坚战。着力解决突出环境问题，促进生态环境质量总体改善。中央财政安排大气、水、土壤三项污染防治资金合计405亿元，比2017年增加64.65亿元，增长19%，投入力度是近年来最大的。支持打赢“蓝天保卫战”，重点支持京津冀等地区大气污染防治，继续开展北方地区冬季清洁取暖试点和秸秆综合利用试点，发展清洁能源。加快水污染防治，实施长江经济带生态修复激励政策，支持中西部污水处理厂提质增效，深入开展蓝色海湾整治行动。强化土壤污染管控和修复，支持土壤污染综合防治先行区示范，推进重金属污染耕地修复治理。实施农村环境综合整治，加强污水、垃圾处理设施建设与维护。实施奖补政策，大力推进畜禽粪污资源化利用。推进“厕所革命”。扩大华北地下水超采区综合治理范围。推动构建以国家公园为主体的自然保护地体系。深入开展山水林田湖草生态保护和修复工程试点。继续实施新一轮草原生态保护补助奖励政策，完善天然林保护制度，扩大退耕还林还草，支持启动大规模国土绿化行动，加强森林资源管护，强化湿地保护与恢复。研究建立多元化、市场化生态补偿机制，积极支持生态环境监管体制改革。
　　2.深化供给侧结构性改革。
　　支持制造业优化升级。综合运用财政专项资金、政府投资基金等方式，支持“中国制造2025”重点领域建设，促进工业与互联网深度融合。继续推动集成电路、新材料等产业发展。降低制造业等行业增值税税率。更好发挥重大技术装备等进口税收政策作用。
　　促进新动能成长壮大。实施“互联网+服务升级”行动，支持传统服务业转型升级和现代服务业加快发展，培育新增长点。延续实施新能源汽车车辆购置税优惠政策，完善补贴政策，加强充电基础设施建设，促进新能源汽车产业做优做强。降低基础电信企业税负，助力新动能成长。
　　继续推进“三去一降一补”。用好中央财政专项奖补资金，支持钢铁、煤炭行业去产能。推动煤电行业化解过剩产能。加大“僵尸企业”债务重组力度，加快市场化法治化债转股步伐。继续推进解决国有企业历史遗留问题、支持中央企业处置“僵尸企业”和治理特困企业工作。在减税降费的同时，健全收费基金项目动态管理机制和乱收费举报投诉查处机制，防止收费项目反弹。发挥投资对优化供给结构的关键性作用，规范推进PPP，加强资本金管理，完善扶持政策体系，鼓励民间资本参与，切实提高公共服务供给质量。
　　3.落实创新驱动发展战略。
　　推动提升科技创新能力。大力支持公共科技活动，加大对基础研究和应用基础研究的投入力度。加快实施国家科技重大专项、科技创新2030-重大项目。支持组建国家实验室和建设一流科研院所。支持科技资源开放共享。进一步落实科研项目资金管理改革等政策，完善中央财政科技计划管理，支持赋予创新团队和领军人才更大的人财物支配权。加快开展面向目标与结果的财政科技支出绩效评价，提高资金使用效益。推动建立以企业为主体、市场为导向、产学研深度融合的技术创新体系，支持符合条件的企业更多承担国家科技计划项目。充分发挥激励机制作用，加速科技成果向现实生产力转化。
　　促进创业创新和小微企业发展。深入开展小微企业创业创新基地城市示范，对拓展小微企业融资担保规模、降低担保费用成效明显的地区给予奖励，促进“双创”升级。设立国家融资担保基金，完善普惠金融发展专项资金政策，优化小微企业、“三农”主体融资环境。
　　4.支持实施乡村振兴战略。
　　完善农业支持保护制度。实施“大专项+任务清单”管理，探索建立涉农资金统筹整合长效机制。完善农业补贴制度，增加产粮大县奖励投入。健全农业信贷担保体系和农业风险分担机制，启动实施三大粮食作物完全成本保险试点。
　　深入推进农业供给侧结构性改革。深化粮食价格形成机制改革，推进稻谷、小麦收储制度改革，完善玉米、大豆市场化收购加补贴机制。继续减少玉米库存，着力消化稻谷库存。扩大耕地轮作休耕制度试点。加大优质粮食工程实施力度。健全绿色农产品生产扶持政策。建立长江流域重点水域禁捕补偿制度。
　　加快发展现代农业。大力推进高标准农田建设，发展高效节水灌溉，加强灾后水利薄弱环节建设。支持农业科技创新，推动重点品种良种培育、关键农机研发。支持多种形式适度规模经营，健全农业生产社会化服务体系，促进小农户和现代农业发展有机衔接。支持发展地方特色优势主导产业，开展农业全产业链开发创新示范，促进农村一二三产业融合发展。推进农垦、国有林区林场等改革发展。
　　深化农村综合改革。落实村级组织经费运转保障机制。扶持村集体经济组织发展。推进美丽乡村建设提档升级，更好发挥一事一议财政奖补机制和平台作用，开展农村综合性改革试点试验。
　　同时，建立健全实施乡村振兴战略财政投入保障制度，加大对农村教育、医疗卫生、文化、基础设施、生态环保等各方面的投入，健全城乡融合发展体制机制。建立新增耕地指标和城乡建设用地增减挂钩节余指标跨省域调剂机制，所得收益全部用于巩固脱贫攻坚成果和支持实施乡村振兴战略。
　　5.推动区域协调发展。
　　提高基本公共服务均等化水平。大幅增加中央对地方一般性转移支付，重点增加均衡性转移支付、老少边穷地区转移支付、民生政策托底保障财力补助等。加大重点领域专项转移支付对发展薄弱地区和财政困难地区的支持力度。继续支持资源枯竭城市转型发展。推进落实兴边富民行动。推动省级财政进一步下沉财力，增强省以下政府基本公共服务保障能力。
　　支持实施国家重点规划。加强对京津冀协同发展、长江经济带发展的支持。推进实施共建“一带一路”倡议。支持高起点规划、高标准建设雄安新区。推动中西部地区、东北地区、东部地区发挥优势、更好发展。促进粤港澳大湾区发展。
　　6.提高保障和改善民生水平。
　　支持发展公平优质教育。教育投入继续向农村、中西部、贫困地区和薄弱环节倾斜。巩固落实城乡统一、重在农村的义务教育经费保障机制，改善贫困地区义务教育薄弱学校基本办学条件。实施第三期学前教育行动计划，多渠道增加学前教育资源供给。推进高中阶段教育普及攻坚计划。深入实施现代职业教育质量提升计划，促进产教融合、校企合作。支持加快世界一流大学和一流学科建设，推动地方高校深化改革和内涵式发展。加强教师队伍建设，进一步健全学生资助制度。支持和规范社会力量兴办教育。
　　加强就业和社会保障。落实积极的就业政策，继续安排就业补助资金，促进就业创业。支持大规模开展职业技能培训，统筹做好高校毕业生、农民工、退役军人、就业困难人员等重点群体就业工作。从2018年1月1日起，提高企业和机关事业单位退休人员基本养老金及城乡居民基础养老金水平。落实养老保险制度改革总体方案，出台实施企业职工基本养老保险基金中央调剂制度，推动划转部分国有资本充实社保基金。进一步推进机关事业单位养老保险制度改革。在部分地区开展个人税收递延型商业养老保险试点。稳慎推进工资收入分配制度改革，完善机关事业单位工资和津补贴制度，向艰苦地区、特殊岗位倾斜。按一定比例适当提高城乡居民低保补助水平，适时调整优抚补助标准，加强对特困人员、残疾人等群体的兜底保障。支持做好留守儿童关爱保护工作。
　　推进健康中国建设。巩固破除以药补医成果，持续深化公立医院综合改革，加强全科医生、儿科医生队伍建设，支持提升医疗服务能力，健全医疗卫生服务体系。全面推进城乡居民医保制度整合，将城乡居民基本医疗保险财政补助标准提高40元（其中20元用于提高大病保险保障水平），达到每人每年490元，相应提高个人缴费比例。将基本公共卫生服务项目年人均财政补助标准再提高5元，达到每人每年55元。推动公立医院强化财务预算管理。扩大跨省异地就医住院费用直接结算范围。支持中医药事业传承创新发展。
　　完善住房保障机制。支持加快建立多主体供给、多渠道保障、租购并举的住房制度。继续推进保障性安居工程建设，2018年支持新开工各类棚户区改造580万套，加大对公共租赁住房及其配套基础设施建设的支持力度，将符合条件的新就业无房职工、外来务工人员纳入公共租赁住房保障范围。继续支持各地优先开展4类重点对象危房改造，进一步加大对深度贫困地区的倾斜力度。
　　推动文化繁荣兴盛。完善公共文化服务体系，深入实施文化惠民工程。继续支持博物馆、图书馆、文化馆等公益性文化设施向社会免费开放。加强文物保护利用和文化遗产保护传承。加强传统媒体和新兴媒体深度融合。支持发展社会主义文艺。促进文化产业发展。推动中华文化走出去。支持做好北京冬奥会、冬残奥会筹办工作。
　　支持平安中国建设。深化司法体制综合配套改革，健全社会治安防控体系，有效维护公共安全。加强社会弱势群体法律援助工作。
　　支持国防和军队现代化建设，为实现强军梦提供有力支撑。继续支持国防和军队改革。积极支持武警部队调整改革。深化国防科技工业投入改革。深入贯彻军民融合发展战略，研究完善配套政策，落实相关资金保障。
　　中央基建投资安排5376亿元，比2017年增加300亿元，加大对“三农”、创新驱动、生态环保、民生改善、安全保障能力建设等领域的支持。优化存量结构，减少小、散支出，提高资金使用绩效。
　　（四）2018年一般公共预算收入预计和支出安排。
　　1.中央一般公共预算。
　　中央一般公共预算收入85357亿元，比2017年执行数同口径（下同）增长5.2%。加上从中央预算稳定调节基金调入2130亿元，从中央政府性基金预算、中央国有资本经营预算调入323亿元，收入总量为87810亿元。中央一般公共预算支出103310亿元，增长8.5%。收支总量相抵，中央财政赤字15500亿元，与2017年持平。中央预算稳定调节基金余额2536.05亿元。中央财政国债余额限额156908.35亿元。
　　中央一般公共预算支出，分中央本级支出、对地方转移支付、对地方税收返还、中央预备费反映。
　　（1）中央本级支出32466亿元，增长8.1%。其中：一般公共服务支出1453.88亿元，增长11.6%，主要是为增强中央垂直管理部门履职能力，适当提高公用经费水平。外交支出600.7亿元，增长15.6%。国防支出11069.51亿元，增长8.1%。公共安全支出1991.1亿元，增长5.5%。教育支出1711.22亿元，增长6.5%。科学技术支出3114.84亿元，增长10.1%。粮油物资储备支出1371.5亿元，下降14.1%，主要是政策性粮棉油去库存力度加大，相应减少利息费用补贴支出。债务付息支出4286.52亿元，增长13.4%。2018年中央部门预算是按照现有机构设置和职能编制的，将根据机构改革情况，在预算执行中依据现有预算规模依法依规作相应调整。
　　（2）对地方转移支付62207亿元，增长9%，增幅为2013年以来最高。
　　对地方一般性转移支付38994.5亿元，增长10.9%，增强地方特别是中西部地区财力。其中：均衡性转移支付24438.57亿元，增长9.1%（含县级基本财力保障机制奖补资金2462.79亿元，增长10%）；老少边穷地区转移支付2133.33亿元，增长15.8%；基本养老金转移支付6696.56亿元，增长14.3%；城乡居民医疗保险转移支付2807.91亿元，增长11.8%。
　　对地方专项转移支付23212.5亿元，增长6.1%。其中：现代职业教育质量提升计划专项资金187.3亿元，增长5.6%；支持地方高校改革发展资金367.32亿元，增长7.8%；就业补助资金468.78亿元，增长6.8%；退役安置补助经费482.19亿元，增长21.2%；困难群众救助补助资金1396.34亿元，增长5.3%；优抚对象补助经费443.62亿元，增长9.7%；公共卫生服务补助资金629.26亿元，增长7.2%；医疗救助补助资金221.13亿元，增长56.7%，主要是支持深度贫困地区提高贫困人口医疗救助水平；农业资源及生态保护补助资金243.6亿元，增长7.6%；中央财政城镇保障性安居工程专项资金1433亿元，增长7.1%。
　　（3）对地方税收返还8137亿元。
　　（4）中央预备费500亿元，与2017年预算持平。预备费执行中视情况分别计入中央本级支出和对地方转移支付。
　　2.地方一般公共预算。
　　地方一般公共预算本级收入97820亿元，增长7%。加上中央对地方税收返还和转移支付收入70344亿元、地方财政调入资金400亿元，地方一般公共预算收入总量为168564亿元。地方一般公共预算支出176864亿元，扣除上年使用结转结余及调入资金后增长7.3%。地方财政赤字8300亿元，与2017年持平，通过发行地方政府一般债券弥补。地方政府一般债务余额限额123789.22亿元。
　　3.全国一般公共预算。
　　汇总中央和地方预算，全国一般公共预算收入183177亿元，增长6.1%。加上调入资金2853亿元，收入总量为186030亿元。全国一般公共预算支出209830亿元（含中央预备费500亿元），扣除上年地方使用结转结余及调入资金后增长7.6%。赤字23800亿元，与2017年持平。赤字率随国内生产总值增长而适当降低，预计为2.6%，比2017年预算降低0.4个百分点，与经济稳中向好、财政运行健康的状况相适应，也为长远发展和宏观调控留下更多政策空间。
　　（五）2018年政府性基金预算收入预计和支出安排。
　　中央政府性基金收入3863.04亿元，增长0.2%。加上上年结转收入385.59亿元，中央政府性基金收入总量为4248.63亿元。中央政府性基金支出4247.17亿元，增长15%。其中，中央本级支出3262.71亿元，增长20.5%，主要是铁路建设基金、民航发展基金、彩票公益金等安排支出增加较多；对地方转移支付984.46亿元，下降0.1%。向一般公共预算调出1.46亿元。
　　地方政府性基金本级收入60301.81亿元，增长4.6%，其中国有土地使用权出让收入54661.7亿元，增长5%。加上中央政府性基金对地方转移支付收入984.46亿元、地方政府专项债务收入13500亿元，地方政府性基金相关收入为74786.27亿元。地方政府性基金相关支出74786.27亿元，增长28.9%，其中国有土地使用权出让收入相关支出66932.08亿元，增长29.3%，主要是对应的专项债务收入大幅增加，相应增加支出安排。地方政府专项债务余额限额86185.08亿元。
　　汇总中央和地方预算，全国政府性基金收入64164.85亿元，增长4.3%。加上上年结转收入385.59亿元和地方政府专项债务收入13500亿元，全国政府性基金相关收入总量为78050.44亿元。全国政府性基金相关支出78048.98亿元，增长28.5%。
　　（六）2018年国有资本经营预算收入预计和支出安排。
　　中央国有资本经营预算收入1376.82亿元，增长5.9%。加上上年结转收入113.59亿元，中央国有资本经营预算收入总量为1490.41亿元。中央国有资本经营预算支出1168.87亿元，增长16.4%。其中，中央本级支出1068.87亿元，增长39%，主要是为支持国有经济结构调整和前瞻性战略性产业发展，适当增加中央企业注资支出；对地方转移支付100亿元，下降57.5%，主要是中央下放企业“三供一业”分离移交补助资金减少。向一般公共预算调出321.54亿元，增长3.6%，调入比例由2017年的22%提高到25%。
　　地方国有资本经营预算本级收入1460.84亿元，增长9.5%。加上中央国有资本经营预算对地方转移支付收入100亿元，地方国有资本经营预算收入1560.84亿元，下降0.6%。地方国有资本经营预算支出1204.71亿元，下降3.2%。向一般公共预算调出356.13亿元。
　　汇总中央和地方预算，全国国有资本经营预算收入2837.66亿元，增长7.7%。加上上年结转收入113.59亿元，全国国有资本经营预算收入总量为2951.25亿元。全国国有资本经营预算支出2273.58亿元，增长12.9%。向一般公共预算调出677.67亿元。
　　（七）2018年社会保险基金预算收入预计和支出安排。
　　根据机关事业单位养老保险改革进展，2018年开始编制中央和地方机关事业单位基本养老保险基金预算，社会保险基金预算首次按中央预算和地方预算分别编制。
　　中央社会保险基金收入676.34亿元，增长97.4%。其中，保险费收入318.61亿元，财政补贴收入352.9亿元。中央社会保险基金支出654.83亿元，增长103.9%。本年收支结余21.51亿元，年末滚存结余270.43亿元。
　　地方社会保险基金收入67416.65亿元，增长22.5%。其中，保险费收入48188.87亿元，财政补贴收入16631.18亿元。地方社会保险基金支出63887.49亿元，增长31.4%。本年收支结余3529.16亿元，年末滚存结余76719.85亿元。
　　2018年中央和地方社会保险基金收支增长较多，主要是将机关事业单位基本养老保险基金纳入预算编制范围。
　　汇总中央和地方预算，全国社会保险基金收入68092.99亿元，增长23%。其中，保险费收入48507.48亿元，财政补贴收入16984.08亿元。全国社会保险基金支出64542.32亿元，增长31.8%。本年收支结余3550.67亿元，年末滚存结余76990.28亿元。
　　需要说明的是，地方预算由地方各级人民政府编制，报同级人民代表大会批准，目前尚在汇总中，本报告中地方收入预计数和支出安排数均为中央财政代编。
　　以上预算具体安排详见《中华人民共和国2017年全国预算执行情况2018年全国预算（草案）》。
　　根据[预算法](https://www.pkulaw.com/chl/0b971dd30ea57e0ebdfb.html)规定，预算年度开始后，在全国人民代表大会批准本预算草案前，可安排下列支出：上年度结转支出；参照上年同期的预算支出数额安排必须支付的本年度部门基本支出、项目支出，以及对下级政府的转移性支出；法律规定必须履行支付义务的支出，以及用于自然灾害等突发事件处理的支出。根据上述规定，2018年1月中央一般公共预算支出11291亿元。其中，中央本级支出1631亿元，对地方税收返还和转移支付9660亿元。

　　**三、**切实做好2018年财政改革发展工作
　　（一）进一步贯彻实施好[预算法](https://www.pkulaw.com/chl/0b971dd30ea57e0ebdfb.html)。
　　增强预算法治意识，更加严格实施[预算法](https://www.pkulaw.com/chl/0b971dd30ea57e0ebdfb.html)，切实硬化预算约束。出台修改后的[预算法实施条例](https://www.pkulaw.com/chl/089bf3d5ca379aecbdfb.html)，加快健全[预算法](https://www.pkulaw.com/chl/0b971dd30ea57e0ebdfb.html)配套制度。推动预算编制的科学化和标准化，完善政府收支分类科目，进一步做实部门预算项目库，完善预算支出定额标准，发挥好预算评审作用，充实和完善预算编制内容。加强年度预算与中期财政规划相衔接，与部门职责和工作安排相匹配，增强预算的科学性、合理性、准确性。规范预算之间的衔接关系，将未列入政府性基金目录清单的项目按规定转列一般公共预算统筹使用，继续提高国有资本经营预算调入一般公共预算的比例，避免资金交叉安排。进一步推进预算公开，扩大向人大报送和社会公开的重点支出项目范围，加大监督检查力度，指导和推动地方做好预算公开工作。继续配合推进预算联网监督。做好向全国人大常委会报告财政医疗卫生资金分配和使用管理情况及接受专题询问工作。
　　（二）加快财税体制改革。
　　出台基本公共服务领域中央与地方共同财政事权和支出责任划分改革方案，研究制定交通运输、科技、环保等领域财政事权和支出责任划分改革方案。抓紧制定中央与地方收入划分改革方案。深化转移支付制度改革，继续清理规范转移支付项目。完善支持农业转移人口市民化的财政政策体系。继续完善增值税制度，结合改革进程推进增值税立法工作。推进综合与分类相结合的个人所得税制度改革，合理提高基本减除费用标准（即提高个人所得税起征点），增加子女教育、大病医疗等专项费用扣除。做好个人收入和财产信息系统建设工作。健全地方税体系，稳妥推进房地产税立法。落实税收法定原则，加快推进单行税法的立法工作。扩大政府财务报告编制试点范围。建立健全国有资产报告机制，推进落实金融企业国有资产专项报告和国有资产综合报告相关工作。加强改革督察，推动改革举措落地见效。
　　（三）全面实施绩效管理。
　　制定全面实施绩效管理的指导性文件。逐步将绩效管理涵盖所有财政资金，并深度融入预算编制、执行、监督全过程。加强重大政策和项目成本效益分析，优化财政资源配置和部门支出结构。强化预算绩效目标管理，优化绩效目标设置，完善绩效目标随同预算批复下达机制。扩大绩效评价范围，建立绩效评价结果与预算安排、政策调整挂钩机制，强化绩效责任硬约束，削减低效无效资金。加大绩效信息公开力度，推进绩效目标、绩效评价结果向人大报送并向社会公开。研究建立地方财政综合绩效评价体系，推动地方财政绩效管理水平整体提升。
　　（四）加强预算执行管理。
　　严格执行人大审查批准的预算，非经法定程序，不得调整。必须作出并需要进行预算调整的，应当报本级人大常委会审查批准。坚持严格依法征税，坚决防止收“过头税”等违规行为。规范和加强非税收入管理，切实降低企业非税负担，进一步推进非税收入法治化建设。完善转移支付分配办法，加强预算批复下达管理，加快转移支付下达进度，促进财政资金及时安排使用并发挥效果。加快盘活财政存量资金以及当年难以支出的预算资金，按规定统筹调整用于其他急需资金支持的领域，减少结转结余。严格执行国库集中收付、政府采购和公务卡制度，规范财政专户资金管理，严禁违规将财政资金从国库转入财政专户。进一步完善国债收益率曲线，加强国债市场建设。加强财政库款管理，研究建立财政库底目标余额管理制度。从严控制新设政府投资基金，分类清理整合现有政府投资基金，防止过多过滥。
　　（五）强化地方政府债务管理。
　　遵循经济规律，举债要同偿债能力相匹配。开好合法合规举债的“前门”，合理确定分地区地方政府债务限额，稳步推进专项债券管理改革，丰富专项债券品种，合理扩大专项债券使用范围，研究开展地方政府债务管理绩效评价。严堵违法违规举债的“后门”，决不允许在法定限额外违法违规或变相举债。精准制定应对预案，积极稳妥化解地方政府债务风险。督促金融机构审慎合规授信，严格按照企业项目实际，而不是按照政府信用评估融资风险。进一步优化地方债市场化发行管理机制，统筹运用银行间和证券交易所两个市场，推动在商业银行柜台销售地方政府债券，提高地方政府债券流动性。推进地方政府融资平台公司市场化转型。加大督查问责力度，完善政绩考核体系，坚决查处各类违法违规融资担保行为，从严整治举债乱象，终身问责、倒查责任。
　　（六）进一步严肃财经纪律。
　　坚持厉行节约，勤俭办一切事业，严禁铺张浪费、大手大脚花钱。加大财政监督检查力度，推动重大财税改革和财税政策落地实施。重点关注扶贫、养老等领域以及对企业补助资金的管理使用情况，坚决查处各种虚报冒领、截留挪用等违反财经纪律的行为，强化责任追究。进一步深化财政内控工作，加强内控执行，着力构建覆盖财政资金分配、管理、使用全流程的内控机制。加大审计问题整改力度，建立健全整改长效机制。
　　主动接受人大依法监督和政协民主监督，既是财政部门的一项法定职责，更是一项重要政治任务。过去的一年，财政部加强全国“两会”财政解释说明服务，强化与代表委员的日常沟通联络，顺利完成2780件全国人大代表建议、1363件全国政协委员提案的办理答复。新的一年，财政部将为代表委员提供更加精细化、规范化的服务，及时报告财税改革和财政重点工作进展，着力提高建议提案办理质量，坚持不懈地转变作风，不断提高预算管理水平和财政治理能力，更好服务代表委员依法履职。
　　各位代表，新时代呼唤着新气象新作为。我们要更加紧密地团结在以习近平同志为核心的党中央周围，高举中国特色社会主义伟大旗帜，以习近平新时代中国特色社会主义思想为指导，自觉接受全国人大的监督，虚心听取全国政协的意见和建议，锐意进取，埋头苦干，扎扎实实做好财政预算各项工作，促进经济社会持续健康发展，为实现党的十九大确定的宏伟目标不懈奋斗！

第十三届全国人民代表大会财政经济委员会关于2017年中央
和地方预算执行情况与2018年中央和地方预算草案的审查结果报告
（2018年3月15日第十三届全国人民代表大会第一次会议主席团第五次会议通过）

十三届全国人大一次会议主席团：
　　第十三届全国人民代表大会第一次会议审查了国务院提出的2017年全国预算执行情况与2018年全国预算草案和《关于2017年中央和地方预算执行情况与2018年中央和地方预算草案的报告》。在第十二届全国人民代表大会财政经济委员会对预算草案和预算报告进行初步审查的基础上，第十三届全国人民代表大会财政经济委员会根据各代表团的审查意见，又作了进一步审查。国务院根据审查意见对预算报告作了修改。现将审查结果报告如下。

　　**一、**根据国务院报告的2017年中央和地方预算执行情况，全国一般公共预算收入172566亿元，完成预算的102.3%，比2016年同口径（下同）增长7.4%，加上使用结转结余及调入资金10139亿元，收入总量为182705亿元；全国一般公共预算支出203330亿元，完成预算的104.3%，增长7.7%，加上补充中央预算稳定调节基金3175亿元，支出总量为206505亿元；收支总量相抵，赤字23800亿元，与十二届全国人大五次会议批准的预算持平，赤字率为2.9%。其中，中央一般公共预算收入81119亿元，完成预算的103.2%，增长7.1%，加上调入中央预算稳定调节基金1350亿元，从中央政府性基金预算、中央国有资本经营预算调入283亿元，收入总量为82752亿元；中央一般公共预算支出95077亿元，完成预算的99.3%，增长5.4%，加上补充中央预算稳定调节基金3175亿元，支出总量为98252亿元；收支总量相抵，中央财政赤字15500亿元，与预算持平。中央预算稳定调节基金余额4666亿元。2017年末，中央财政国债余额134770.16亿元，控制在全国人大批准的债务余额限额141408.35亿元之内。地方政府债务余额164706.59亿元，包括一般债务余额103322.35亿元、专项债务余额61384.24亿元，控制在全国人大批准的债务余额限额188174.3亿元以内。
　　全国政府性基金收入61462亿元，完成预算的130.3%，增长34.8%，加上结转收入299亿元和地方政府发行新增专项债券筹集收入8000亿元，全国政府性基金收入总量为69761亿元；全国政府性基金支出60700亿元，完成预算的109.4%，增长32.7%。其中，中央政府性基金收入3824亿元，完成预算的103.2%，增长6.4%，加上结转收入299亿元，中央政府性基金收入总量为4123亿元；中央政府性基金支出3669亿元，完成预算的91.6%，增长9.2%。全国国有资本经营预算收入2579亿元，完成预算的110.3%，下降1.2%；全国国有资本经营预算支出2011亿元，完成预算的102.5%，下降6.7%，向一般公共预算调出568亿元。其中，中央国有资本经营预算收入1244亿元，完成预算的96.5%，下降13%，加上结转收入128亿元，收入总量为1372亿元；中央国有资本经营预算支出1002亿元，完成预算的86.3%，下降30.9%，向一般公共预算调出257亿元。全国社会保险基金收入55380亿元，完成预算的106.9%，增长10.5%；全国社会保险基金支出48952亿元，完成预算的101%，增长12.3%；当年收支结余6428亿元，年末滚存结余72037亿元。预算草案中对执行情况进行了说明。
　　财政经济委员会认为，2017年中央和地方预算执行总体情况较好。面对复杂严峻的国内外环境，在以习近平同志为核心的党中央坚强领导下，国务院和地方各级人民政府不断增强“四个意识”，认真学习贯彻习近平新时代中国特色社会主义思想和党的十九大精神，按照党中央决策部署和十二届全国人大五次会议各项决议的要求，贯彻实施[预算法](https://www.pkulaw.com/chl/0b971dd30ea57e0ebdfb.html)，坚持稳中求进工作总基调，贯彻落实新发展理念，以推进供给侧结构性改革为主线，实施积极的财政政策，加大减税降费力度，着力保障脱贫攻坚、生态环保、民生改善等重点领域支出，深化财税体制改革，促进了经济社会持续健康发展，发挥了财政在国家治理中的基础和重要支柱作用。同时，在预算执行和财政管理中还存在一些不容忽视的问题，主要是：财政收支仍处于紧平衡状态，部分基层财政运行困难；预算编制还不够科学准确，预算执行的约束力不够强，支出项目只增不减的固化格局没有根本改变，部分项目执行结果与预算相差较大；一些政策尚未执行到位，一些资金使用绩效不高，存在资金闲置、项目推进缓慢、违规收取部分行政事业性收费问题；部门预算执行中存在通过项目支出列支公用经费和人员经费、年末结转资金规模较大等现象；转移支付管理不够规范，部分专项转移支付项目预算执行率偏低；一些地方存在变相举债和违法违规担保行为，地方政府隐性债务风险不容忽视。这些问题要高度重视，认真研究，采取有效措施切实加以解决。

　　**二、**国务院提出的2018年中央和地方预算草案，全国一般公共预算收入183177亿元，比2017年预算执行数（同口径，下同）增长6.1%，加上调入资金2853亿元，收入总量为186030亿元；全国一般公共预算支出209830亿元，扣除上年地方使用结转结余及调入资金后增长7.6%；收支总量相抵，赤字23800亿元，与上年持平。其中，中央一般公共预算收入85357亿元，增长5.2%，从中央预算稳定调节基金调入2130亿元，从中央政府性基金预算、中央国有资本经营预算调入323亿元，收入总量为87810亿元；中央一般公共预算支出103310亿元，增长8.5%；收支总量相抵，中央财政赤字15500亿元，与上年持平；中央财政国债余额限额156908.35亿元。地方政府一般债务余额限额123789.22亿元。
　　全国政府性基金收入64165亿元，增长4.3%，加上上年结转收入386亿元、地方政府专项债券收入13500亿元，收入总量为78051亿元；全国政府性基金支出78049亿元，增长28.5%。其中，中央政府性基金收入3863亿元，增长0.2%，加上上年结转收入386亿元，收入总量为4249亿元，中央政府性基金支出4247亿元，增长15%。地方政府专项债务余额限额86185.08亿元。全国国有资本经营预算收入2837亿元，增长7.7%，加上上年结转收入114亿元，收入总量为2951亿元；全国国有资本经营预算支出2274亿元，增长12.9%，向一般公共预算调出678亿元。其中，中央国有资本经营预算收入1377亿元，增长5.9%，加上上年结转收入114亿元，收入总量为1491亿元；中央国有资本经营预算支出1169亿元，增长16.4%，向一般公共预算调出322亿元。全国社会保险基金收入68093亿元，增长23%；全国社会保险基金支出64542亿元，增长31.8%；本年收支结余3551亿元，年末滚存结余76990亿元。其中，中央社会保险基金收入676亿元，增长97.4%；中央社会保险基金支出655亿元，增长103.9%；本年收支结余21亿元，年末滚存结余270亿元。
　　财政经济委员会认为，国务院提出的2018年中央和地方预算草案，符合党的十九大和中央经济工作会议精神，贯彻落实党中央重大方针政策和决策部署，坚持稳中求进工作总基调，坚持新发展理念，紧扣我国社会主要矛盾变化，按照高质量发展要求，坚持以供给侧结构性改革为主线，继续实施积极的财政政策，进一步优化财政支出结构，支持打好三大攻坚战，推动解决发展不平衡不充分的问题，促进经济社会持续健康发展。预算草案符合[预算法](https://www.pkulaw.com/chl/0b971dd30ea57e0ebdfb.html)的规定。预算草案总体可行。
　　财政经济委员会建议，第十三届全国人民代表大会第一次会议批准国务院提出的《关于2017年中央和地方预算执行情况与2018年中央和地方预算草案的报告》，批准2018年中央预算草案，同时批准地方政府一般债务余额限额123789.22亿元、地方政府专项债务余额限额86185.08亿元。地方各级政府预算依法由本级人民代表大会审查和批准。各省、自治区、直辖市人民政府依照国务院下达的债务限额举借的债务，列入本级预算调整方案，报本级人大常委会依法批准。国务院将地方预算汇总后报全国人民代表大会常务委员会备案。

　　**三、**2018年是全面贯彻党的十九大精神的开局之年，是改革开放40周年，是决胜全面建成小康社会、实施“十三五”规划承上启下的关键一年，做好财政预算工作意义重大。要在以习近平同志为核心的党中央坚强领导下，以马克思列宁主义、毛泽东思想、邓小平理论、“三个代表”重要思想、科学发展观、习近平新时代中国特色社会主义思想为指导，全面深入贯彻党的十九大和十九届二中、三中全会精神，坚持和加强党的全面领导，扎实做好各项财政预算工作，圆满完成2018年预算，促进经济社会持续健康发展。为此，财政经济委员会提出以下建议：
　　（一）继续实施积极的财政政策。继续实施减税降费，提高减税降费政策的精准性针对性，进一步优化纳税服务，全面落实各项税收优惠政策，切实增强市场主体的获得感。清理规范各类政府性基金和行政事业性收费项目，取消不合法的收费项目。以供给侧结构性改革为主线，继续推进“三去一降一补”，支持制造业优化升级。优化财政支出结构，提高财政支出的公共性和普惠性。财政资金要向“三农”倾斜，健全投入保障机制，加强顶层设计，加快建立涉农资金统筹整合长效机制，支持实施乡村振兴战略。坚持精准扶贫、精准脱贫，新增扶贫资金要向深度贫困地区倾斜，着力解决深度贫困地区公共服务、基础设施欠缺等问题。加大教育、医疗卫生、社会保障等民生保障投入。加快缩小地区间基本公共服务差距，增强困难地区和基层政府保工资、保运转、保基本民生的能力。要树立过紧日子的思想，严肃财经纪律，采取有效措施，压缩一般性支出，厉行勤俭节约。
　　（二）认真落实重要改革举措。适应人大预算审查监督重点向支出预算和政策拓展的改革要求，预算决算报告、草案应重点反映贯彻落实党中央重大方针政策和决策部署情况、重大支出政策和税收政策调整变化情况、重大投资项目安排和实施情况等。做好2018年向全国人大常委会提交国有资产管理情况综合报告和专项口头报告金融企业国有资产管理情况有关工作。要主动听取人大代表和社会各界意见建议，积极回应各方关切。推进审计管理体制改革，健全完善审计查出问题整改的长效机制。
　　（三）全面实施[预算法](https://www.pkulaw.com/chl/0b971dd30ea57e0ebdfb.html)。加快修订并出台[预算法实施条例](https://www.pkulaw.com/chl/089bf3d5ca379aecbdfb.html)。加强财政收入预测，依法组织收入，提高收入质量，严禁收入虚增、空转等行为。树立预算法治意识，严格按照全国人民代表大会批准的预算执行，增强预算的严肃性和约束力。根据机构改革方案，依法做好相关部门预算的批复和变更工作。中央对地方转移支付资金要依法尽早细化到项目和地方，并及时下达。提高预算编制的科学性，完善基本支出定员定额管理，加快推进项目支出标准体系建设。全面实施预算绩效管理，将绩效管理理念、方法和要求嵌入预算管理全过程，提高资金使用绩效。依法严格规范结转结余资金管理。进一步加大预算、决算公开力度。
　　（四）切实防范地方政府隐性债务风险。要采取果断措施，坚决遏制地方政府隐性债务增量。要抓紧摸清地方政府隐性债务底数，积极稳妥化解隐性债务存量。有关地方要制定应急预案，建立市场化、法治化债务违约处置机制。要剥离融资平台公司政府融资职能，推进地方政府融资平台市场化转型。研究完善地方政府新增债务限额的确定和管理机制。健全监督问责机制，从严整治地方举债乱象，坚决查处违法违规融资行为，终身问责、倒查责任。
　　（五）深化财税体制改革。积极推动中央与地方财政事权和支出责任划分改革，落实基本公共服务领域共同财政事权和支出责任划分改革方案，加快制定教育、医疗卫生、交通运输等重点领域财政事权和支出责任划分改革方案。尽快研究制定中央与地方收入划分改革总体方案，推动健全地方税体系。做好税收征管体制改革工作。规范转移支付分配和管理。国有资本经营预算中要反映国有企业的运营情况。2018年建立基本养老保险基金中央调剂制度。加快落实划转部分国有资本充实社保基金工作。加快政府会计标准体系建设，积极推进编制政府综合财务报告工作。
　　（六）加强税收立法工作。2020年前完成落实税收法定原则的改革任务，时间十分紧迫、任务非常艰巨。有关部门要高度重视税收立法工作，加大工作力度，采取有效措施，确保如期完成改革任务。2018年要及时将耕地占用税、车辆购置税、资源税等税收法律草案和[税收征收管理法](https://www.pkulaw.com/chl/ca88b5ed5ce08c6cbdfb.html)（修改）草案，提请全国人大常委会审议。加快推动综合与分类相结合的个人所得税制改革。做好加快房地产税立法并适时推进改革的相关工作。加快推进增值税等税种立法工作。推进非税收入法治化进程。
　　以上报告，请予审议。

第十三届全国人民代表大会财政经济委员会
2018年3月15日

© 北大法宝：（[www.pkulaw.com](https://www.pkulaw.com/)) 专业提供法律信息、法学知识和法律软件领域各类解决方案。北大法宝为您提供丰富的参考资料，正式引用法规条文时请与标准文本核对。
欢迎查看所有[产品和服务](https://www.pkulaw.com/helps/2.html)。法宝快讯：[如何快速找到您需要的检索结果？](https://www.pkulaw.com/helps/70.html)[法宝](https://www.pkulaw.com/helps/69.html)V6有何新特色？

扫描二维码阅读原文

**原文链接：**<https://www.pkulaw.com/chl/f23509d0e80fcf5dbdfb.html>
